# The cannabinoid receptors agonist WIN55212-2 inhibits macrophageal differentiation and alters expression and phosphorylation of cell cycle control proteins

**DOI:** 10.1186/1478-811X-9-33

**Published:** 2011-12-28

**Authors:** Katrin Paulsen, Svantje Tauber, Johanna Timm, Nadine Goelz, Claudia Dumrese, Alexandra Stolzing, Ralf Hass, Oliver Ullrich

**Affiliations:** 1 Division of Cellbiology, Institute of Anatomy, Faculty of Medicine, University of Zurich, Switzerland; 2 Center for Microsocopy and Image Analysis, University of Zurich, Switzerland; 3 Stem Cell Biology Group, Fraunhofer Institute for Cell Therapy and Immunology, Leipzig, Germany; 4 Biochemistry and Tumor Biology Lab, Department of Gynecology, Medizinische Hochschule Hannover, Germany; 5 Zurich Center for Integrative Human Physiology (ZIHP), University of Zurich, Switzerland

**Keywords:** Immune control, Macrophages, Differentiation, Cannabinoids

## Abstract

In this study we investigated if and how cannabinoid receptor stimulation regulates macrophageal differentiation, which is one of the key steps in the immune effector reaction. For that reason, we used a well established differentiation model system of human U937 myelocytic leukemia cells that differentiate along the monocyte/macrophage lineage upon stimulation with the phorbol ester PMA. Constant cannabinoid receptor (CB) stimulation was performed using WIN55212-2, a potent synthetic CB agonist. We found that WIN55212-2 inhibited CB1/2-receptor-dependent PMA-induced differentiation of human myelocytic U937 cells into the macrophageal phenotype, which was associated with impaired vimentin, ICAM-1 and CD11b expression. In the presence of WIN55212-2, cdc2 protein and mRNA expression was progressively enhanced and Tyr-15-phosporylation of cdc2 was reduced in differentiating U937 cells. Additionally, p21^Waf1/Cip1 ^expression was up-regulated. PMA-induced apoptosis was not enhanced by WIN55212-2 and differentiation-associated c-jun expression was not altered. In conclusion, we suppose that WIN55212-2-induced signals interferes with cell-cycle-arrest-signaling in differentiating myelocytic cells and thus inhibits macrophageal differentiation. Thus, it is possible that the cannabinoid system is able to influence one of the key steps in the immune effector function, the monocytic-macrophageal differentiation by alteration of cell cycle control proteins cdc2 and p21, and is therefore representing a promising option for therapeutic intervention in exacerbated immune reactions.

## Introduction

Cannabinoids have been used as medicinal plant extracts for a long time. Already 4000 years ago, the Chinese emperor successfully treated diseases associated with increased immune reaction and inflammation using plant extracts [1]. The fact that the signaling pathways of the cannabinoid system are conserved throughout various species suggests an evolutionary benefit [2,3]. Cannabinoid signals can be mediated by different receptors, the first cannabinoid receptor CB1 was discovered in 1990 in the brain. A few years later a second receptor CB2 was cloned from immune cells [4,5]. The activation of CB1 and CB2 results in different cellular responses: (i) inhibition of adenylyl cyclase and the cAMP/protein kinase A (PKA)-dependent pathway by inhibitory G-proteins (Gi) which leads to a reduced production of cAMP [6] while recent research suggests that these receptors can also stimulate cAMP production by directly stimulating G-proteins (Gs) [7] (ii) stimulation of mitogen-activated protein kinase (MAPK) cascade, especially the extracellular signal kinase (ERK) [8] and the p38 MAPK cascade [9]. Whereas the CB1 receptor is mainly, but not exclusively, expressed on neurons, the CB2 receptor is primarily present in immune cells [8,10]. Other receptors in the cannabinoid signal system include the vanilloid receptor type 1 (TRPV1) [11] and the G protein-coupled receptor 55, also known as GPR55 [12,13]. Interestingly, many cannabinoid-related compounds have little if any affinity for either of the two known cannabinoid receptors CB1 or CB2 - suggesting that other unidentified receptors might be involved. The endogenous ligands for the endocannabinoid system are anandamide (AEA), 2-Arachidonylglycerol (2-AG), noladin ether and virodhamine, but fast degradation of these substances by the specific monoglyceride lipase (in the case of 2-AG), serine hydrolase and fatty acid amide hydrolase (for AEA and 2-AG) limits the usage of these ligands for the study of the signaling pathways [14,15]. Therefore stable, non-degradable synthetic cannabinoids such as CP55940, HU210 (CB1) and WIN55212-2 (CB1/CB2) are often used for the analysis of signaling pathways and molecules [16,17]. In our study, we used WIN55212-2 ((R)-(+)-[2, 3-Dihydro-5-methyl-3-(4-morpholinylmethyl)pyrrolo[1, 2, 3-de)-1, 4-benzoxazin-6-yl]-1-napthalenylmethanone mesyalate), a potent aminoalkylindole cannabinoid (CB) receptor agonist for human CB1 and CB2 receptors, in micromolar concentrations as described in other cellular studies [18].

Different cannabinoids have been shown to influence the functional activities of various immune cells in rodents as well as in humans, including B lymphocytes, T lymphocytes, natural killer cells and macrophages [18-22]. In these studies, cannabinoids demonstrated pleiotropic effects on cells of the immune system such as suppressed T cell proliferation, inhibited antibody response, enhanced B cell growth, various effects on macrophages and their functionality as well as inhibition of cytokines and a switch from inflammatory to anti-inflammatory cytokine subtypes [17,21]. Recently, it was shown that CB2 mediated immunosuppressive activities of AEA in primary T lymphocytes, including Th-17 cells, an important finding in the light of new endocannabinoid-based immunotherapeutic approaches [19]. CB_2 _receptors are widely expressed in cells of the immune system such as in monocytes/macrophages, B cells, NK cells, and T cells [20-22]. General mechanisms by which cannabinoid receptor activation is considered to act immunosuppressive (for a review see [21] are for example 1.) decreasing the expression of cAMP-responsive genes by inhibition of cAMP/protein kinase A (PKA) pathway, 2.) enhancement of apoptotic genes regulated by NF-kappaB by phosphorylation of IkappaB-alpha, 3.) PPARgamma-dependent inhibition of NF-AT activation or 4.) interference in the cell cycle by activation of p21^waf-1/cip-1 ^and induction of a G0/G1 phase arrest. Cannabinoids seem to control immune function by interfering at different points and key mechanisms of the orchestrated immunological network: CB2-mediated inhibition of TNF-alpha, IL-1beta, IL-6 and IL-8 release by monocytes/macrophages, Th1- to Th2-type cytokine shift in T cells, as wells as suppression of IFN-γ and IL-12 release.

Cells of the monocyte-macrophage-system constantly patrol the organism, transmigrate trough the blood vessels and into the tissues, where they differentiate into the macrophageal phenotype, a phagocytosing immunoeffector cells, which also presents antigens towards T cells. Cannabinoid receptor agonists are known to suppress macrophage functions such as phagocytosis, bactericidal activity, NO production and cell migration [21,23-25].. However, it is still not known if and how cannabinoid receptor stimulation regulates macrophageal differentiation, which is one of the key steps in the immune effector reaction. For that reason, we used a well established differentiation model system of human U937 myelomonocytic leukemia cells that allows investigation of differentiation-dependent alterations [26-29]. Treatment of U937 cells with phorbol-12-myristate-13-acetate (PMA) is accompanied by growth arrest and induction of a differentiation program, in which the cells acquire a variety of morphological and functional changes associated with macrophages.

## Materials and methods

### Cell culture

U937 (human, myelocytes) cells were obtained from American Tissue and Cell Culture (ATCC) and cultured in RPMI-1640 with 10% of fetal calf serum (FCS) and 1% of stable glutamine (all Biochrom, Germany) at 37°C under normoxia (air plus 5% CO_2_) in a humidified atmosphere. Cells were seeded at a density of 0.2 × 10^6 ^cells/ml and were subcultured upon reaching 1, 0 × 10^6 ^cells/ml. Medium was changed every 2 to 3 days. Cells were counted using Neubauer chamber and trypan blue (Sigma-Aldrich) was used for viability determination.

### Differentiation

For differentiation of U937 myelocytic cells into a monocyte/macrophage-like phenotype, the cells were treated with 25 nM phorbol-12-myristate-13-acetate (PMA) (Sigma-Aldrich) for 72 hours at a densitiy of 0.5 × 10^6 ^cells/ml. PMA was diluted in DMSO (Sigma-Aldrich), the concentration of DMSO was 1:1000 at all times in every group. Control cells were incubated without PMA, but in 1:1000 DMSO. The share of differentiated cells was measured at the time points of 24, 48 and 72 hours. Cells were collected by gently scraping (cell scraper) in culture medium, centrifuged for 8 min at 300 g and the supernatant was removed. Cell pellets were washed twice in ice cold PBS and directly analyzed (viability, flow cytometer) or stored at -80°C for further analysis (immunoblotting, qRT-PCR). Optimal PMA concentration was selected by determining the percentage of differentiated cells and their viability. In preliminary experiments, viability of U937 cells has been tested in the presence of PMA concentrations up to 200 nM and of WIN55212-2 concentrations up to 10 μM. Treatment of U937 cells with PMA results in a decrease of viability in the non-adherent cell fraction after 2 h, whereas WIN55212-2 exhibited no toxicity up to a concentration of 5 μM and 72 h incubation (viability > 90%). Inhibition of cannabinoid type 1 (CB1) and type 2 (CB2) receptors was performed by co-incubation with 2 μM AM251 (CB1-inhibition) and/or 2 μM AM630 (CB2-inhibition).

### Viability Assays (MTT and annexin V/propidium iodide FACS)

Cytotoxicity was measured by 3-(4, 5-dimethylthiazol-2-yl)-2, 5- diphenyltetrazolium bromide (MTT) assay and annexin V/propidium iodide (PI) staining for apoptosis/necrosis. Different WIN55212-2 concentrations (2-10 μM) and 25 nM PMA were added simultaneously or separately to the cell culture. Viability (MTT-assay) and percentage of apoptosis/necrosis (flow cytometry analysis; annexin V/propidium iodide) were measured at different time points (30 min-72 h). Phosphatidylserine exposure and propidium iodide incorporation was assessed using the annexinV-FLUOS staining kit (Roche) following manufacturer's instructions. U937 cells were treated separately with PMA or simultaneously with PMA/WIN55212-2 and gently scraped in medium afterwards. Cells were collected and washed in PBS followed by 15 min staining with annexin V/propidium iodide solution at room temperature and analyzed by flow cytometry (Becton Dickinson, Basel, Switzerland). For immunocytochemical analysis of apoptotic cells, cells were stained with annexin V and DAPI after fixation with 3% paraformaldehyde and 2% sucrose in PBS and analyzed by microscopy.

### Staining and FACS analysis of ICAM-1 and CD11b

Different WIN55212-2 concentrations (3-5 μM), 25 nM PMA and CB1 (2 μM AM251) or CB2 (2 μM AM630) inhibitor were added simultaneously or separately to the cell culture. U937 cells were treated separately with PMA, simultaneously with PMA/WIN55212-2 or simultaneously with PMA/WIN55212-2 and AM251 or AM630 and gently scraped in RPMI-1640 medium afterwards. Cells were collected and washed in PBS followed by 30 min staining with ICAM-1-PE (BD Bioscience), CD11b-APC (BD Bioscience) and SYTOX AADvanced (Invitrogen; staining of dead cells, dead cells were not calculated). Staining procedure was according to the manufacturer's protocols. Cells were analyzed immediately after staining by flow cytometry (FACS Canto II, Becton Dickinson, Basel, Switzerland).

### Detection of cell cycle control proteins by immunoblot analysis

Total protein lysates were produced with an ice cold hypotonic lysis buffer composed of Tris (50 mM), EDTA (10 mM), Na_3_VO_4 _(1 mM), NaF (10 mM), NaCl (165 mM), 1 mM PMSF, with 1% Triton-X 100 and 1 mM protein inhibitor cocktail (Sigma-Aldrich). Cells were incubated for 15 min in lysis buffer on ice, followed by 25s sonication at 50% intensity. Lysates were continuously shaken for 10 min and then centrifuged at 14, 000 rpm for 10 min at 4°C. Pellets were removed and protein concentration was determined using BCA protein kit (Pierce). Supernatants were stored at -80°C. Aliquots of lysates were incubated in 50% glycerol (Sigma-Aldrich), 5% 2-mercaptoethanol (Fluka), and 0.5% bromophenol blue (Sigma-Aldrich), boiled for 5 min and centrifuged shortly. Proteins were separated in 12% SDS-polyacrylamide gels (Fluka). Each lane was loaded with 40 μg protein extract. Separated proteins were transferred in 20% Tris-methanol buffer onto a nitrocellulose membrane (Pierce) and blocked with Li-Cor blocking buffer (Biochrom) diluted 1:1 with PBS (Biochrom) for 2 hours. Membranes were stored between filter papers at -20°C. After thawing, membranes were washed in TBS-Tween (0, 1%) for 10 min (Sigma-Aldrich) and then incubated with polyclonal antibodies against cdc2, cdc2^Tyr15^, cdc2^Thr161^, chk1, p-chk1, c-jun, GAPDH (all from Cell Signaling) as well as actin and vimentin (both from Sigma) overnight at 4°C using dilutions as described in the appropriate datasheets. Specific binding of the primary antibodies was detected by corresponding secondary infrared dye-conjugated anti-mouse IgG antibodies, incubated at room temperature for 2 hours in Li-Cor blocking buffer (Li-Cor) 1:1 diluted with PBS (Biochrom) and visualized by an Odyssey infrared imaging system (Li-Cor Biosciences). Intensities were calculated as percentage of band signals detected by the Li-Cor system relative to non-induced control cells. For normalization between different blot membranes, an internal control (S0) has been used. Additional staining of the membranes with actin and GAPDH, dependent of the size of the primary detected protein, was used as loading control.

### Vimentin-, Actin- and DAPI-staining for microscopic analysis

U937 cells were treated with PMA or with PMA/WIN55212-2 and gently scraped in medium afterwards. Cells were collected and washed in PBS followed by fixation with 3% paraformaldehyde and 2% sucrose in PBS and attached onto glass slides by cytospin. Samples were permeabilized with 0.2% Triton-X 100 in PBS for 5 min at RT followed by incubation with rabbit-anti-vimentin antibody (1:200, Abcam) in 2% BSA in PBS at 4°C overnight. Cells were then incubated with donkey-anti rabbit FITC-labelled antibody at a 1:200 dilution (Jackson immune research), phalloidin-texas red (1:50) (molecular probes) and 1 μg/ml DAPI (Molecular probes) in PBS for 1 h at RT. Samples were then embedded in fluorescence mounting medium (Dako) and analyzed using a confocal laser scanning microscope (SP2, Leica, Heidelberg, Germany).

### Quantitative real-time PCR analysis (qRT-PCR)

Total RNA was extracted from 2-5 × 10^6 ^cells using the RNeasy Mini Kit Isolation System (Qiagen, Hombrechtikon) and DNA was removed using the RNase-Free DNase Set (Qiagen, Hombrechtikon) according to the manufacturer's protocols. One hundred ng of RNA was transcribed with SuperScript First-Strand Synthesis System for RT-PCR (Invitrogen,) in a 20 μl reaction volume according to the manufacturer's instruction for First-Strand cDNA Synthesis using Oligo(dT). The relative expression of genes was determined using FAM-labeled TaqMan Gene Expression Assays (Applied Biosystems) for cdc2 (Hs00938777_m1), p21 (Hs99999142_m1), CB1 (Hs01038522_s1), CB2 (Hs00361490_m1) and glyceraldehyde-3-phosphate dehydrogenase (GAPDH) as housekeeping gene expression control (Hs99999905_m1). Quantitative real-time PCR (qRT-PCR) was performed using 0.2 μl of cDNA in a 20 μl reaction volume containing 5 μl TaqMan Gene Expression Master Mix in MicroAmp Fast Optical 96-Well Reaction Plates. Samples were run on an ABI 7500 Fast Real-Time-PCR system (Applied Biosystems), thermal cycling conditions were 50°C for 2 min followed by 95°C for 10 min and 40 cycles of 95°C for 15 s and 60°C for 1 min. The relative quantification of gene expression was performed using the comparative CT method (DDCt). All reagents and devices for quantitative real time PCR were purchased from Applied Biosystems (Rotkreuz, Switzerland).

### Statistical analysis

For statistical analysis the non-parametric Mann-Whitney test was used. All experiments were performed in minimum in triplicate at three different time points at three independent days (n = 3, triplets). p < 0.05 was set as significance level.

## Results

### WIN55212-2 inhibited differentiation of human monocytic U937 cells into an adherent phenotype

To study the effect of cannabinoid receptor stimulation on macrophageal differentiation, we used a well characterized differentiation system of human myelocytic U937 cells [26-29], which can be induced by phorbol ester to differentiate into adherent macrophage-like cells paralleled by a cell cycle arrest of the autonomously proliferating tumor cells.. For this purpose, proliferating U937 cells were incubated with PMA alone or together with WIN55212-2. Differentiation was tracked by determining the share of differentiated and cell cycle-arrested adherent cells and the share of non-differentiated proliferating suspended cells at defined points from 24 h up to 72 h after addition of PMA and/or WIN55212-2 by FACS counting.

**Figure 1:**
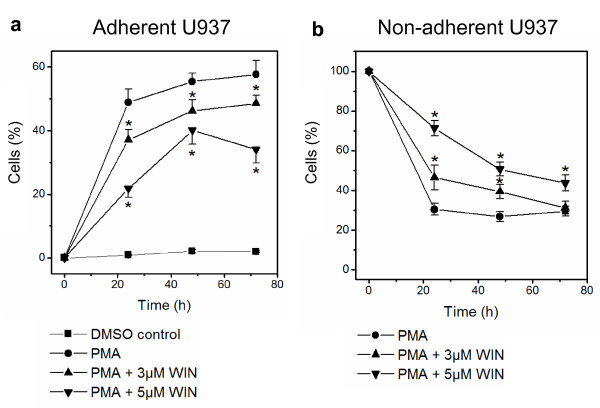
**WIN55212-2 inhibited differentiation of human monocytic U937 cells into an adherent phenotype**. Differentiation of U937 cells was induced by 25 nM PMA with or without co-incubation with WIN55212-2. The experimental groups were: 1.) Vehicle control = treatment with 0.1% DMSO only, 2.) Differentiation control = treatment with 25 nM PMA, 3.) Experimental groups were treated additionally with 3 μM or 5 μM WIN55212-2, respectively. Adherent (figure 1a) and non-adherent cells (figure 1b) were separated and quantified after different time points up to 72 h by FACS analysis. Percentage of control group at time point zero is shown. Data are shown as mean +/- SEM, n = 12; *p < 0.05 in comparison with the PMA-treated group without WIN55212-2.

PMA clearly induced U937 cell differentiation with a pronounced increase of adherent cells within the first 24 h (Figure 1a). In the presence of WIN55212-2, PMA-induced differentiation of U937 cells was severely impaired and adherent cells were reduced to nearly half of the non-treated group during all time points (Figure 1a). In parallel to the differentiation process, non-adherent U937 cells disappeared continuously from the culture medium (Figure 1b). Consistently with the decelerated adhesion of differentiating U937 cells after PMA-induction, the additionally WIN-exposed U937 cells persisted longer and in higher concentrations in the cell culture supernatant (Figure 1b).

### Inhibition of macrophageal differentiation of human U937 cells by WIN55212-2 was CB1- and CB2-receptor-dependent

**Figure 2:**
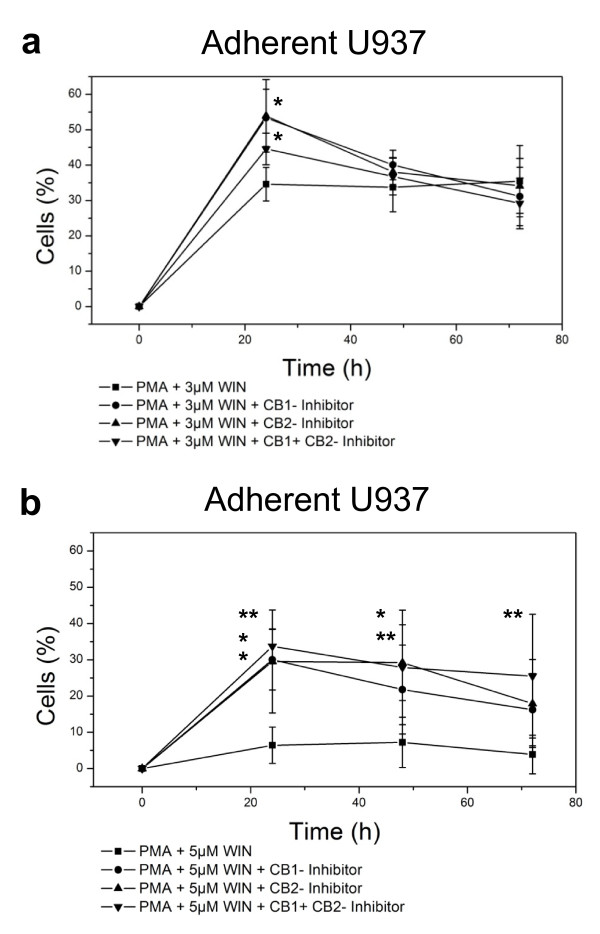
**WIN55212-2-mediated inhibition of differentiation of human monocytic U937 cells is CB1- and CB2-receptor-dependent**. Differentiation of U937 cells was induced by 25 nM PMA in the presence of 3 μM or 5 μM WIN55212-2 (figure 2a or figure 2b, respectively) with or without co-incubation with 2 μM AM251 (CB1-inhibition) and/or 2 μM AM630 (CB2-inhibition). Adherent cells were separated and quantified after different time points up to 72 h by FACS analysis. Percentage of control group at time point zero is shown. Data are shown as mean +/- SD, n = 8; *p < 0.05, **p < 0.01, ***p < 0.001 in comparison with the PMA/WIN55212-2 -treated group without CB1- or CB2-inhibition.

We investigated, if the inhibition of PMA-induced macrophageal differentiation of U937 cells was dependent on cannabinoid receptor type 1 (CB1) or type 2 (CB2) activity and incubated PMA/WIN55212-treated U937 cells with the CB1 receptor inhibitor AM251 and/or the CB2 receptor inhibitor AM630. Both CB1 and CB2 receptor inhibition restored the differentiation process as demonstrated by a significant increase of adherent cells after 24 h incubation with PMA/3 μM WIN55212 (Figure 2a) and a significant increase of adherent cells after 24-72 h incubation with PMA/5 μM WIN55212 (Figure 2b). Thus, the inhibitory action of WIN55212-2 on macrophageal differentiation of U937 cells is CB1- and CB2-receptor-dependent.

### WIN55212-2 did not enhance PMA-induced apoptosis

**Figure 3:**
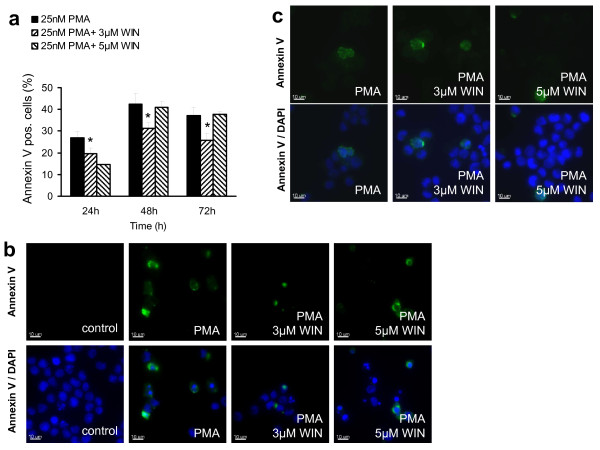
**WIN55212-2 did not enhance PMA-induced apoptosis**. Differentiation of U937 cells was induced by 25 nM PMA with or without co-incubation with WIN55212-2. The experimental groups were: 1.) Vehicle control = treatment with 0.1% DMSO only, 2.) Differentiation control = treatment with 25 nM PMA, 3.) Experimental groups were treated additionally with 3 μM or 5 μM WIN55212-2, respectively. Differentiated (a) and non-differentiated cells (b) were separated due to its adherence after 72 h incubation and apoptotic cells were detected by annexin V staining and FACS analysis (figure 3a) or immunocytochemical analysis after counterstaining with DAPI (figure 2b). Annexin V-positive cells in the non-differentiated cell fraction are quantified in figure 3a and displayed in figure 3b. Immunocytochemical analysis of adherent cells are shown in figure 3c. Data are shown as mean +/- SEM, n = 12; *p < 0.05 in comparison with the total cell number. Scale bar = 10 μm.

Since it is well known that cannabinoid receptor stimulation may induce apoptosis in different cell types of the immune system [21], we first investigated whether incubation with WIN55212-2 drives proliferating U937 cells into apoptosis upon induction of differentiation by PMA, therefore subsequently reducing the number of differentiated cells. As demonstrated in Figure 3a, WIN55212-2 did not enhance PMA-induced apoptosis in U937 cells, but in contrast protected significantly non-differentiated cells from apoptosis at a concentration of 3 μM at all time points (Figure 3a). Additional immunocytochemical analysis of U937 cells after 72 h incubation with PMA alone or in the presence of WIN55212-2 showed less annexin V-positive cells in the WIN55212-2-cotreated group (Figure 3b), which supported also visually the results of the FACS analysis (Figure 3a). In the DMSO control group, no annexin V positive apoptotic cells could be detected. Annexin V-staining of adherent cells revealed only a few apoptotic cells in this differentiated U937 cell fraction (Figure 3c). FACS analysis after propidium iodide staining demonstrated no differences in all cell fractions, but only a slightly higher percentage of propidium iodide positive cells in the PMA-differentiated U937 cell fraction, most probably due to cell scraping during harvest (data not shown).

### WIN55212-2 impaired vimentin expression in PMA-induced U937 cells

**Figure 4:**
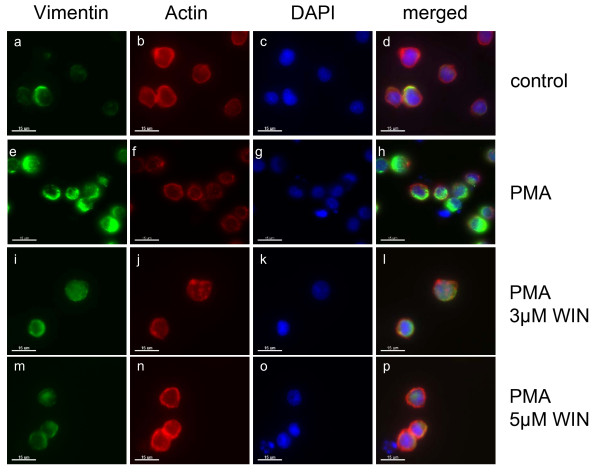
**Immunocytochemical analysis of vimentin expression in PMA-induced U937 cells co-incubated with WIN55212-2**. Differentiation of U937 cells was induced by 25 nM PMA with or without co-incubation with WIN55212-2. The experimental groups were: 1.) Vehicle control = treatment with 0.1% DMSO only (a, b, c, d), 2.) Differentiation control = treatment with 25 nM PMA (e, f, g, h), 3.) Experimental groups were treated additionally with 3 μM (I, j, k, l) or 5 μM (m, n, o, p) WIN55212-2, respectively. Cells were stain against vimentin (a, e, i, m), actin (b, f, j, n) and DAPI (c, g, k, o) and analysed by confocal laser scanning microscopy. Merged pictures are shown (d, h, l, p). Scale bar = 15 μM.

**Figure 5:**
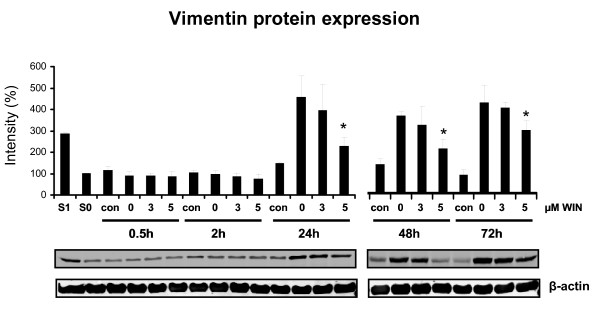
**Immunoblot analysis of vimentin protein expression in PMA-induced U937 cells co-incubated with WIN55212-2**. Differentiation of U937 cells was induced by 25 nM PMA with or without co-incubation with WIN55212-2. The experimental groups were: 1.) Vehicle control = treatment with 0.1% DMSO only **(con)**, 2.) Differentiation control = treatment with 25 nM PMA **(0)**, 3.) Experimental groups were treated additionally with 3 μM **(3) **or 5 μM **(5) **WIN55212-2, respectively. Vimentin protein expression was analysed by immunoblotting and quantified by Odyssey infrared imaging system (Li-Cor Biosciences) and shown as mean +/- SEM, n = 3. Relative intensities were normalized to a non-induced control (S0) and a PMA-induced control (S1) which served as internal control for quantification if different blot membranes have been used. *p < 0.05. One representative blot and one representative beta-actin control out of 3 is shown.

One of the most prominent alterations during macrophageal differentiation of U937 cells is the reorganization of the cytoskeleton. Thus, we investigated cytoskeletal changes by actin- and vimentin staining after 72 h incubation with PMA with/without WIN55212-2 (Figure 4). Whereas actin expression and intracellular distribution was not altered in all groups, expression of vimentin was enhanced distinctly after PMA treatment compared to the controls, but was ameliorated by co-incubation with 3 μM or 5 μM WIN55212-2. Immunoblot analysis of vimentin expression in PMA-induced U937 cells during different time points of differentiation demonstrated a significant reduction of vimentin protein expression between 24 h to 72 h after the induction of differentiation (Figure 5). As soon as vimentin protein rised during the differentiation process after 24 h, its increasing expression was ameliorated in WIN55212-2-treated U937 cells. Since vimentin is highly expressed during macrophageal differentiation [29], the findings support an inhibitory effect of cannabinoid receptor stimulation on macrophageal differentiation and cytoskeletal reorganization.

### WIN55212-2 did not alter differentiation-associated c-jun expression

**Figure 6:**
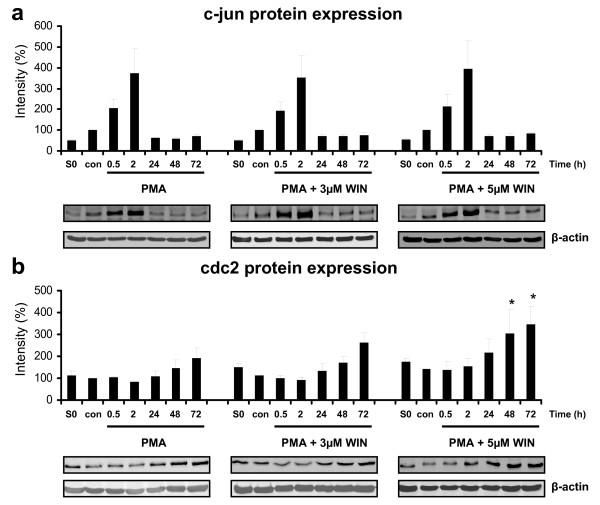
**Immunoblot analysis of c-jun and cdc2 protein expression in PMA-induced U937 cells co-incubated with WIN55212-2**. Differentiation of U937 cells was induced by 25 nM PMA with or without co-incubation with WIN55212-2. The experimental groups were: 1.) Vehicle control = treatment with 0.1% DMSO only **(con)**, 2.) Differentiation control = treatment with 25 nM PMA **(0)**, 3.) Experimental groups were treated additionally with 3 μM **(3) **or 5 μM **(5) **WIN55212-2, respectively. C-jun (figure 5a) and cdc2 (figure 5b) protein expression was analysed by immunoblotting and quantified by Odyssey infrared imaging system (Li-Cor Biosciences) and shown as mean +/- SEM, n = 3. Relative intensities were normalized to non-induced control cells (con) and an internal control (S0) for quantification if different blot membranes have been used. *p < 0.05. One representative blot and one representative beta-actin control out of 3 is shown.

Macrophageal differentiation is associated with a complex regulation of different signal pathways and the expression of differentiation-related transcription factors, such as c-jun, which forms together with c-fos the AP-1 early response transcription factor, which in turn controls a number of signal processes including differentiation, proliferation, and apoptosis. C-jun is therefore considered as one of the key molecules and an early and rapid response marker of macrophageal differentiation [28,29]. We therefore analysed c-jun protein expression by immunoblotting, which was enhanced 4-fold after 2 h of PMA treatment (Figure 6a), much earlier than the cellular reorganization commenced (Figure 4 and 5). C-jun protein expression then returned to nearly control levels after 24 h. No differences in c-jun protein expression could be detected in dependence of WIN55212-2-treatment (Figure 6a). Thus, WIN55212-2-mediated effects on macrophageal differentiation are likely independent from c-jun expression.

### WIN55212-2 altered expression and phosphorylation of cell cycle control proteins

Induction of U937 cells differentiation with PMA results predominantly in a G_0_/G_1 _and partly in a G_2_/M cell cycle arrest without any detectable S phase cells [29-31]. For transition through the G_2_/M phase, phosphorylation and dephosphorylation of different signaling molecules, such as cdc2 and the cyclin dependent kinase 1 (cdk1) are necessary. Because the entry into mitosis is regulated by cdc2 kinase activation, which is controlled crucially by dephosphorylation of cdc2 at Tyr15 and phosphorylation at Thr161 [32], we investigated the effect of WIN55212-2 on cdc2 protein expression and phosphorylation by immunoblotting. As demonstrated in Figure 6b, cdc2 protein expression increased between 24 h and 72 h after induction of differentiation. In the presence of 5 μM WIN55212-2, cdc2 protein expression upon PMA-treatment was enhanced significantly (Figure 6b).

**Figure 7:**
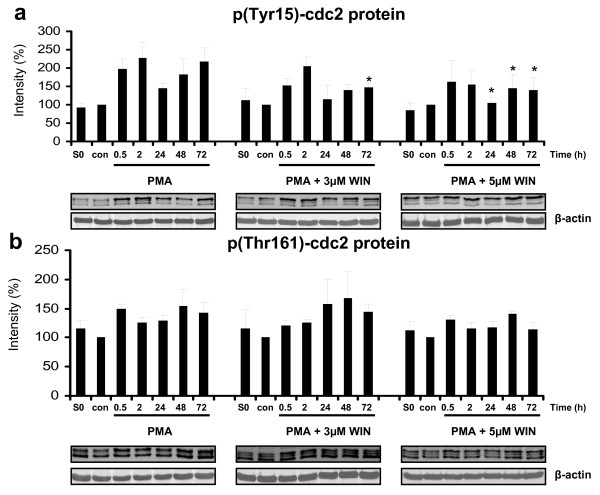
**WIN55212-2 altered phosphorylation of cdc2 protein**. Differentiation of U937 cells was induced by 25 nM PMA with or without co-incubation with WIN55212-2. The experimental groups were: 1.) Vehicle control = treatment with 0.1% DMSO only **(con)**, 2.) Differentiation control = treatment with 25 nM PMA **(PMA)**, 3.) Experimental groups were treated additionally with 3 μM **(PMA + 3 μM WIN) **or 5 μM **(PMA + 5 μM WIN) **WIN55212-2, respectively. Tyr15-cdc2 (figure 6a) and Thr161-cdc2 (figure 6b) phosphorylation was analysed by immunoblotting and quantified by Odyssey infrared imaging system (Li-Cor Biosciences) and shown as mean +/- SEM, n = 3. Relative intensities were normalized to non-induced control cells (con) and an internal control (S0) for quantification if different blot membranes have been used. *p < 0.05. One representative blot and one representative beta-actin control out of 3 is shown.

During macrophageal differentiation, cdc2 was strongly phosphorylated at Tyr15 (Figure 7a), which prevents cells from entering into mitosis and contributes to cell cycle arrest. In the presence of WIN55212-2, Tyr15-phosphorylation of cdc2 was significantly and constantly reduced between 24 h and 72 h after induction of differentiation (Figure 7a). Additionally, Thr161-phosphorylation was not altered during PMA-induced macrophageal differentiation and not influenced by WIN55212-2 (Figure 7b).

**Figure 8:**
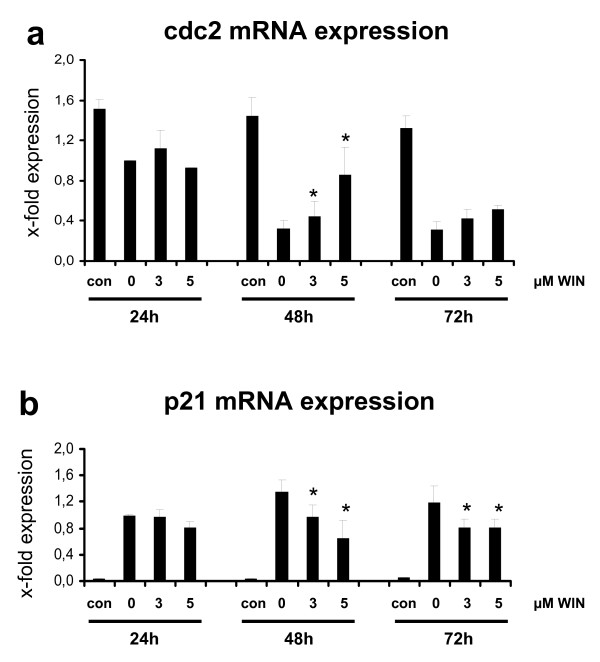
**Quantitative real time PCR analysis of cdc2 and p21 mRNA expression in PMA-induced U937 cells co-incubated with WIN55212-2**. Differentiation of U937 cells was induced by 25 nM PMA with or without co-incubation with WIN55212-2. The experimental groups were: 1.) Vehicle control = treatment with 0.1% DMSO only **(con)**, 2.) Differentiation control = treatment with 25 nM PMA **(0)**, 3.) Experimental groups were treated additionally with 3 μM **(3) **or 5 μM **(5) **WIN55212-2, respectively. cdc2 (figure 7a) and p21 (figure 7b) mRNA expression were analysed after different time points by quantitative real-time PCR. Data are shown as mean +/- SEM, n = 3. Relative expression were normalized to GAPDH expression in non-induced cells. *p < 0.05.

In order to investigate whether the enhanced cdc2 protein concentration is the result of an increased cdc2 transcription, we analyzed cdc2 mRNA concentration in PMA-induced U937 cells in the presence of absence of incubation with WIN55212-2. As demonstrated in Figure 8a, PMA-induced downregulation of cdc2 mRNA expression was significantly ameliorated during additional WIN55212-2-treatment of U937 cells.

Additionally, we determined the mRNA expression of the cell cycle regulatory protein p21^Waf1/Cip1^, which forms heterotrimeric complexes with cyclins and cyclin-dependent kinases, inhibits CDK2 kinase activity and blocks progression before the G1/S cell cycle checkpoint. PMA induced a dramatic increase of p21^Waf1/Cip1 ^mRNA expression in U937 cells which is consistent with differentiation-associated cell cycle arrest. In the presence of WIN55212-2, up-regulation of p21^Waf1/Cip1 ^was distinctly ameliorated (Figure 8b). Taken together, WIN55212-2 significantly altered the expression and phosphorylation of PMA-induced cell cycle control proteins such as cdc2 and p21 (Figure 8ab).

### No downregulation of CB1 and CB2 mRNA expression by WIN55212-2

**Figure 9:**
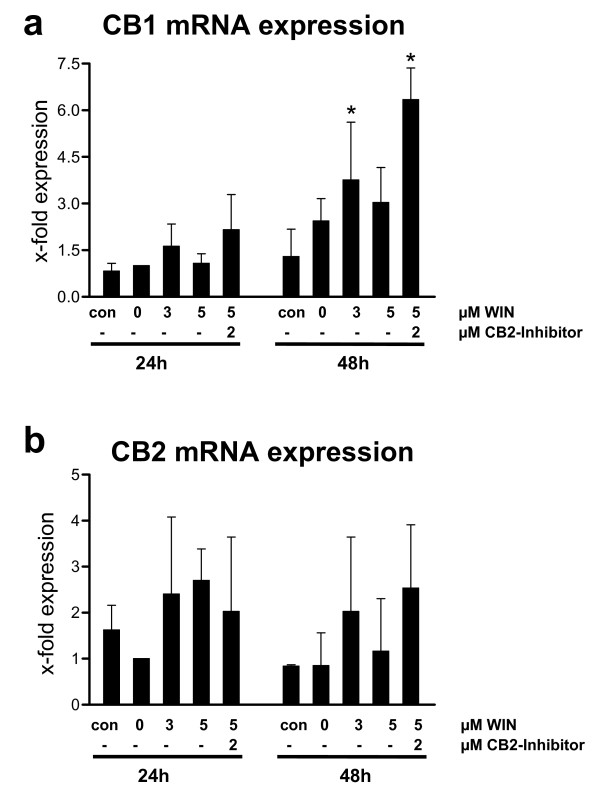
**Quantitative real time PCR analysis of CB1 and CB2 receptor mRNA expression in PMA-induced U937 cells co-incubated with WIN55212-2**. Differentiation of U937 cells was induced by 25 nM PMA with or without co-incubation with WIN55212-2. The experimental groups were: 1.) Vehicle control = treatment with 0.1% DMSO only **(con)**, 2.) Differentiation control = treatment with 25 nM PMA **(0)**, 3.) Experimental groups were treated additionally with 3 μM **(3) **or 5 μM **(5) **WIN55212-2, respectively. One group was treated with 5 μM **(5) **WIN55212-2 and 2 μM WIN AM630 (CB2 inhibition). CB1 (figure 9a) and CB2 (figure 9b) receptor mRNA expression were analysed after different time points by quantitative real-time PCR. Data are shown as mean +/- SD, n = 3. Relative expression were normalized to GAPDH expression in non-induced cells. *p < 0.05.

Because feedback-regulation is a common and frequent principle in cells of the immune system, we investigated the influence of WIN55212-2 on CB1 and CB2 expression during PMA-induced macrophageal differentiation of U937 cells (Figure 9). We detected an increase in CB1 mRNA expression in the presence of WIN55212-2, which was enhanced by parallel inhibition of CB2 receptors (Figure 9a). The detected changes in CB2 mRNA expression were not significant (Figure 9b). Also, the elevation of CB1 mRNA in differentiating U937 cells was not significant compared to undifferentiated controls (Figure 9a).

### CB1 and CB2 receptor-dependent down-regulation of ICAM-1 and CD11b in WIN55212-2 treated differentiating U937 cells

**Figure 10:**
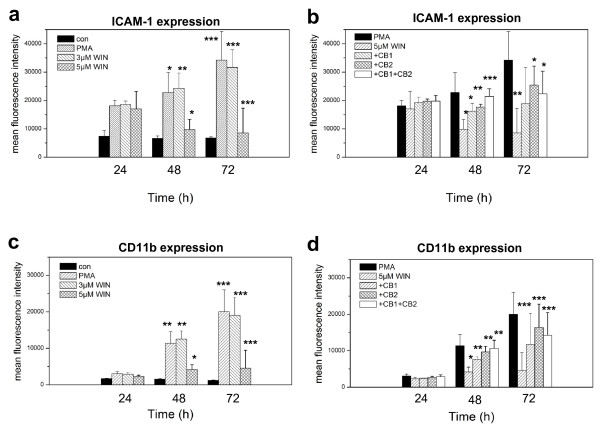
**FACS analysis of ICAM-1 and CD11b expression in PMA-induced U937 cells co-incubated with WIN55212-2**. Differentiation of U937 cells was induced by 25 nM PMA with or without co-incubation with 3 μM or 5 μM WIN55212-2 with or without 2 μM AM251 (CB1 inhibition) and/or 2 μM AM630 (CB2 inhibition). ICAM-1 (figure 10ab) and CD11b (figure 10cd) expression were measured according to the manufacturer's protocols by flow cytometry.

Finally, we investigated the effect of WIN55212-2 on ICAM-1 and CD11b expression in differentiating U937 cells. We found that ICAM-1 was up-regulated in differentiating U937 cells, which was distinctly reduced by co-incubation with 5 μM WIN55212-2 (Figure 10a). Reduction of differentiation-associated ICAM-1 up-regulation was less pronounced after inhibition of CB1 and CB2 receptors (Figure 10b). The expression of CD11b demonstrated a similar effect: Whereas CD11b was induced in differentiating U937, it was reduced by 5 μM WIN55212-2 (Figure 10c) in a CB1- and CB2-receptor-dependent manner.

## Discussion

In our study we demonstrated that the cannbinoid receptor agonist WIN55212-2 inhibited macrophageal differentiation CB1- and CB2-receptor-dependent in a well established monocyte-macrophageal differentiation model. Inhibition of macrophageal differentiation by WIN55212-2 was associated with impaired vimentin expression, enhanced cdc2 protein and mRNA expression and reduced Tyr-15-phosporylation of cdc2 upon PMA-induction.

Previous studies have shown that the incubation with PMA of human leukemic cell lines like myelocytic U937 (also e.g. monocytic THP1 and promyelocytic HL60) induced a differentiation along a phagocytic lineage. This led to growth inhibition, cell cycle arrest, adherence, expression or upregulation of differentiation-associated molecules like the cytoplasmic intermediate filament vimentin or the transcription factor c-jun, as well as activation and deactivation of different signaling molecules like cdk1, cdk2, c-fos and others [26-29,33,34]. Moreover, it was reported that the morphology and a variety of functional parameters do not significantly change during PMA-induced differentiation until the cells enter a retrodifferentiation program after long term culture [27].. Therefore, influences on differentiation and maturation of U937 cells are likely to take place within the first three days of PMA treatment. Thus, we aimed to investigate the influence of sustained cannabinoid-receptor-stimulation using the synthetic and stable cannabinoid receptor agonist WIN55212-2 on PMA-induced differentiation of human myelocytic U937 cells into a macrophage-like phenotype and to address the changes of molecular architecture possibly involved in this process.

First, we investigated changes in protein expression pattern of the cytoplasmic intermediate filament vimentin, which has a strong influence during the differentiation process to support cell adherence as well as reorganization of cell structure and stabilizes the architecture of the cytoplasm [35]. We found that differentiation-related vimentin-expression was ameliorated by WIN55212-2. C-jun is a component of transcription factor AP-1 (activator protein 1), which activates the TRE/AP-1 transcription element and plays a role in diverse biological functions such as proliferation, differentiation and apoptosis [36-38]. Both molecules are upregulated after PMA treatment in U937 as well as in HL-60 and THP-1 cells [26-29]. Since we detected no differences in c-jun protein expression in dependence of WIN55212-2-treatment, we suppose that cannabinoid-mediated effects on macrophageal differentiation are independent from c-jun expression.

Various studies showed that WIN55212-2 has pro-apoptotic effects and results in growth arrest and downregulation of cell cycle dependent kinases like cdk2, cdk4 and cdk6 in different tumor cell lines such as HepG2 hepatoma cells [31]. In our study, we found that WIN55212-2 did not enhance apoptosis in PMA-treated U937 cells, but in contrast protected non-differentiated U937 cells from apoptosis. Since PMA-induced apoptosis is mediated likely via an autocrine TNF-alpha feedback loop [39] and since it is known that cannabinoid receptor stimulation inhibits TNF-alpha release in monocytic cells [40], the anti-apoptotic effect of WIN55212-2 in PMA-stimulated U937 cells could be mediated via interference with autocrine TNF-alpha secretion.

Because differentiation of U937 requires cell cycle arrest, we investigated expression and phosphorylation of the crucial cell cycle regulatory protein cdc2. Previous studies in U937 cells demonstrated that the regulation of cdc2 is cell-cycle-dependent, undetectable concentrations of cdc2 in G_1 _but maximum expression of mRNA and protein in G_2_/M transition were measured [28]. The transition of eukaryotic cells into M-phase is regulated by cdc2 kinase activation, a process controlled at several steps including cyclin binding together with varying protein levels and phosphorylation of cdc2 at Thr161, Tyr14 and Tyr 15 position. The critical regulatory step is the activation of cdc2 by dephosphorylation at Tyr14 and Tyr15 by the phosphatase cdc25 [32,41-44]. PMA activates the calcium- and phospholipid dependent protein kinase C, but it is still unclear whether the activation of this enzyme contributes directly to the cell cycle regulatory gene expression or if the introduction of the differentiation process is responsible for the downregulation of these genes [45]. The fact that other PKC activators like bryostatin 1 are considered to be a weaker differentiation stimuli compared to PMA in U937, resulting in a smaller p21 activation and cell cycle arrest, strongly supports the second theory [46]. Nevertheless, it could be shown that the expression of cdc2 and cdc25 mRNA is downregulated and cdc2 phosphorylation at Tyr15 position is upregulated upon PMA treatment, thus, resulting in a cell cycle arrest essential for the differentiation process [28]. In our study, we detected an enhanced cdc2 protein and mRNA expression associated with reduced Tyr-15-phosporylation in differentiating U937 cells treated with WIN55212-2.

Additionally, in the presence of WIN55212-2, differentiation-induced up-regulation of p21^Waf1/Cip1 ^is distinctly ameliorated. p21^Waf1/Cip1 ^inhibits G1 cyclins and associated cyclin-dependent kinases whereby induction of p21^Waf1/Cip1 ^in most cell types is accompanied by a G0/G1 cell cycle arrest. Expression of p21 is induced in a p53-dependent manner after DNA damage. However, after direct stimulation of PKC by PMA treatment the elevation of p21 expression has been reported in a p53-independent manner [46,47], which may support the present model because of the non-functional p53 in the U937 cells line. Additionally, in proliferating AGS and MKN-1 gastric cancer cell lines, treatment with WIN 55, 212-2 induced p21^Waf1/Cip1 ^and arrested the cell cycle in the G0/G1 phase, which was mediated via activation of the MAPK pathway and inhibition of pAKT [48]. Because in our experiments, we found an ameliorated. p21^Waf1/Cip1 ^upregulation in PMA-induced differentiating U937 cells, it can be assumed that WIN 55, 212-2 transmitted cell cycle arrest signals, which are dependent on PKC-activation and probably on the cell type and its proliferative state.

Our experiments suggest that stimulation of CB1/2 receptors by WIN55212-2 during macrophageal differentiation did not downregulate CB1 and CB2 mRNA expression and therefore did not activate a negative feedback-loop during differentiation. In contrast, CB1 receptor expression was significantly upregulated in response to WIN55212-2 treatment during macrophageal differentiation. Therefore, we assume that the cannabinoid receptor systems maintains a constant regulatory influence of cannabinoid receptor agonists during macrophageal differentiation. In differentiating macrophageal cells, CD11b and ICAM-1 are predominantly expressed and mediates inflammation by regulating leukocyte adhesion and migration and has been implicated in several immune processes. We found that WIN55212-2 reduced CB1- and CB2-receptor-dependent the expression of CD11b and ICAM-1.

In conclusion, we suppose that WIN55212-2-mediated CB1- and CB2-receptor-dependent signals reduce PMA-mediated cell-cycle-arrest-signaling in maturating cells along the monocytic lineage and therefore inhibit macrophageal differentiation. Thus, it is possible that the cannabinoid system is able to influence one of the key steps in the immune effector function, the monocytic-macrophageal differentiation by alteration of cell cycle control proteins cdc2 and p21, and is therefore representing a promising option for therapeutic intervention in exacerbated immune reactions.

## Abbreviations

CB: Cannabinoid receptor; qRT-PCR: quantitative real-time polymerase chain reaction; PMA: phorbol-12-myristate-13-acetate; WIN55212-2: (R)-(+)-[2, 3-Dihydro-5-methyl-3-(4-morpholinylmethyl)pyrrolo[1, 2, 3-de)-1, 4-benzoxazin-6-yl]-1-napthalenylmethanone mesyalate.

## Competing interests

The authors declare that they have no competing interests.

## Authors' contributions

KP carried out the cell cultures studies and molecular analysis and helped to draft the manuscript. KP also participated in the study design and coordination. JT, ST and NG participated in the cell culture studies and molecular analysis. CD carried out the immunocytochemical analysis. AS helped to draft the manuscript and contributed to the study coordination. RH contributed to the cell cycle analysis and critically edited the manuscript. OU contributed the study concept, design, coordination and supervision and drafted and edited the manuscript. All authors read and approved the final manuscript.
